# Video Calls as a Replacement for Family Visits During Lockdowns in Aged Care: Interview Study With Family Members

**DOI:** 10.2196/40953

**Published:** 2023-06-12

**Authors:** Ryan M Kelly, Yushan Xing, Steven Baker, Jenny Waycott

**Affiliations:** 1 School of Computing and Information Systems University of Melbourne Melbourne Australia; 2 School of Human Services and Social Work Griffith University Brisbane Australia

**Keywords:** aged care, COVID-19 pandemic, lockdowns, older adults, video calls, videoconferencing, mobile phone, COVID-19

## Abstract

**Background:**

Lockdowns have been used to prevent the spread of transmissible illnesses such as influenza, norovirus, and COVID-19 in care homes. However, lockdowns deny care home residents supplemental care and the socioemotional enrichment that comes from seeing family members. Video calling has the potential to enable ongoing contact between residents and family members during lockdowns. However, video calls can be considered by some as a poor substitute for in-person visits. It is important to understand family members’ experiences with video calling during lockdowns to ensure the effective use of this technology in the future.

**Objective:**

This study aimed to understand how family members use video calls to communicate with relatives living in aged care during lockdowns. We focused on experiences during the COVID-19 pandemic, which involved extensive lockdowns in aged care homes.

**Methods:**

We conducted semistructured interviews with 18 adults who had been using video calls with relatives living in aged care during pandemic lockdowns. The interviews focused on how participants had been using video calls, what benefits they gained from video-based interactions, and what challenges they encountered when using the technology. We analyzed the data using the 6-phase reflexive approach to thematic analysis by Braun and Clarke.

**Results:**

We developed 4 themes through our analysis. Theme 1 interprets video calling as a medium for the continuation of care during lockdowns. Using video calls, family members were able to provide social enrichment for residents and engaged in health monitoring to uphold residents’ welfare. Theme 2 highlights how video calling extended care by supporting frequent contact, transmitting nonverbal cues that were essential for communication, and negating the need for face masks. Theme 3 interprets organizational issues such as the lack of technology and staff time as impediments to the continuation of familial care through video. Finally, theme 4 highlights the need for 2-way communication, interpreting residents’ unfamiliarity with video calling and their health conditions as further barriers to the continuation of care.

**Conclusions:**

This study suggests that, during restrictions arising from the COVID-19 pandemic, video calls became a medium for enabling family members to continue participating in the care of their relatives. The use of video calls to continue care illustrates their value for families during times of mandatory lockdown and supports the use of video to complement face-to-face visits at other times. However, better support is needed for video calling in aged care homes. This study also revealed a need for video calling systems that are designed for the aged care context.

## Introduction

### Background

Lockdowns are a common measure for preventing the spread of viral diseases in aged care homes [1,2]. In recent years, lockdowns have been used to slow the spread of COVID-19 in care homes worldwide [3,4]. These lockdowns were essential because of considerable outbreaks of COVID-19 within care facilities [5,6] and because care home residents were highly susceptible to mortality from the disease [7]. Examples of measures implemented during lockdowns include the confinement of residents to their rooms and the suspension of all face-to-face visits from family and friends [8-10].

Although lockdowns can be an effective way to prevent disease transmission, prolonged periods of isolation are highly detrimental to the cognitive and emotional well-being of care home residents [11]. Loneliness and isolation have long been salient problems in aged care [12,13], and there is evidence suggesting that these problems worsened during the pandemic lockdowns [10,14]. This can be partly attributed to the loss of physical visits from residents’ family members, who were not able to visit in person while restrictions were in place [15]. Beyond the positive health and well-being effects of visits from family [16,17], family members have been described as “central to the care of residents” [14]. Families are a primary source of cognitive and emotional enrichment [18] and contribute to advocacy, emotional support, and assistance with the personal care of residents [14,19-21]. The loss of such support during the pandemic restrictions has placed renewed focus on enabling meaningful communication between residents and families during lockdowns [9,22,23].

After the onset of the pandemic, many aged care homes adopted video calling systems as a replacement for face-to-face visits [13,24]. These systems—which include Skype (Skype Technologies), FaceTime (Apple), and Zoom (Zoom Video Communications)—allow 2 or more people to see each other and participate in real-time conversations using high-fidelity video and audio feeds transmitted over the internet [25]. Several studies have used survey methods to understand the availability of video calling hardware during the pandemic [26,27] and whether care homes had to adjust the provision of technology for social connection [13]. These studies provide important data to illustrate care providers’ adoption of video calling but do not offer insights into the experiences of people who had to rely on video calling in lieu of face-to-face visits during the lockdowns.

The need to understand the utility of video calling as a replacement for face-to-face visits, especially during lockdowns, is important for multiple reasons. First, the pandemic was perhaps the first time that video calling was deployed en masse in care homes. There have been studies illustrating the benefits of video calls for aged care residents, including positive impacts on social connection [28], emotional enrichment [29], and feelings of depression and loneliness among residents [30]. However, many such studies have involved short-term trials coordinated by research teams in which residents used video calls with relatives who could not visit the care home [28,31,32]. This setup partially replicates the circumstances of a lockdown, but it may not capture issues and experiences that arise in real-world use when support from researchers is unavailable. The recent lockdowns because of COVID-19 afford an important learning opportunity in this regard given that care homes had to pivot quickly to using the technology without external support from research teams.

Second, studies conducted before and during the pandemic have identified that video calling is affected by challenges in long-term care. A study conducted in Canada by Chu et al [33] found that essential family caregivers were highly dissatisfied with the provision of video calls during the lockdowns. The reasons given included poor internet availability, unsuitable devices, and technical problems, all of which have been identified as barriers in previous work [27,31,34]. Moreover, many aged care residents are frail, have complex health conditions such as dementia, and have little experience with information and communications technologies. These issues may make it hard for them to use commercial video calling systems [35,36], and care staff usually need to provide extensive assistance [37]. However, care staff typically have limited time for activities that fall outside their core duties [27,32], meaning that video calls risk placing additional burdens on an already overstretched workforce [37]. In addition, staff shortages were prevalent during the COVID-19 pandemic owing to high rates of infection within care homes [38]. These issues may have affected the quality of experience for residents and their families, but there remains scant understanding of people’s experiences of video calling as a substitute for in-person visits during the restrictions.

### Objectives

This study aimed to understand how family members used video calls to communicate with relatives living in aged care homes during COVID-19 lockdowns. We focused on experiences of video calling in Australia, where there were extensive and protracted lockdowns that prevented families from visiting relatives during the first 2 years of the pandemic.

This study explored the following questions:

1. How did family members use video calling with relatives in aged care during lockdowns?
2. In what ways was video calling beneficial for family members?
3. What challenges or barriers need to be overcome to support high-quality video calling experiences during lockdowns in aged care?

## Methods

### Ethics Approval

This study was approved by the University of Melbourne Human Research Ethics Committee (ID 1851239.5).

### Study Design

This study used semistructured interviews to investigate family members’ experiences with video calling. We used a semistructured approach to guide each interview using a broad set of questions while enabling participants to bring their own topics and experiences into the conversations [39].

The reflexive thematic analysis (RTA) approach by Braun and Clarke [40,41] was used to analyze and interpret the data. RTA champions the researcher’s interpretation as central to making sense of and generating meaning from qualitative data [41]. This means that RTA does not attempt to define a codebook or demonstrate validity based on metrics such as interrater reliability [42,43]. Rather, the emphasis is on the researcher’s reflexive engagement with data and on interpretation developed through iterative rounds of coding and analysis. The goal of RTA is to develop themes that capture patterns of shared meaning relevant to the research questions [44,45].

Consistent with the assumptions of RTA, we adopted a constructionist and experiential orientation for our data collection and analysis. This means that we prioritized participants’ own accounts of their experiences while using our interpretations to interrogate the meaning within those experiences [43]. We adopted RTA as we did not enter the study with a specific theory or deductive lens through which to interpret the data. RTA provided the flexibility to incorporate relevant theory and use existing knowledge on video calling to inform and develop the evolving analysis [41].

### Research Context

We collected data for this study from July 2021 to October 2021 in the state of Victoria, Australia. Victoria was the site of several major COVID-19 outbreaks in 2020 and 2021. The state government imposed a total of 6 lockdowns that lasted a cumulative 8 months [46]. These lockdowns involved measures such as mandatory social distancing, stay-at-home orders, and the use of face masks both indoors and outdoors [47]. Aged care facilities in Victoria had to adhere to these restrictions, with many imposing bans on visitors because of COVID-19 outbreaks among residents and staff [48]. These restrictions meant that the participants in this study were reliant on video calls as their primary method of seeing relatives during the lockdowns.

### Participant Recruitment

We recruited participants through the University of Melbourne internal staff mailing list and web-based noticeboards, advertisements on Twitter and LinkedIn, and word of mouth. Participants enrolled in the study by contacting the first author via email. There were no preexisting relationships before study commencement.

Our sample size was evaluated throughout the research process [49], and we stopped recruiting after 18 participants had completed the study. In line with RTA, we do not claim this as evidence of data saturation [50]. Rather, we ceased recruitment as the lead researcher (RMK) made the assessment that the data contained sufficient information power to address our aims and research questions [41,49]. Specifically, we felt that no further participants were required as our study had a narrow aim (to understand video calling in aged care); our sample specificity was dense (family members); and participants had conveyed rich experiences, indicating a strong quality of dialogue [49].

### Procedure

Interviews were one-to-one and were conducted by the first author. Before study commencement, we emailed participants a Plain Language Statement containing information about the study’s aims and procedures. Participants also signed a consent form, which they returned to the first author via email.

The researcher met with participants individually using Zoom videoconferencing software. We used Zoom to comply with social distancing requirements, which were in place when our data were collected. We used a semistructured interview guide, with an initial set of open-ended questions that enabled participants to share their personal experiences (see Multimedia Appendix 1 for the questions). The interview questions were developed as a team, drawing on our collective experience of research on digital communication technologies [51,52], older adults [53,54], and aged care [29,55]. The researcher took notes during the interviews and asked follow-up questions to probe responses in detail. The interviews lasted 19 to 47 (mean 32, SD 9) minutes. All interviews were audio recorded with participants’ consent. Each participant received an Aus $30 (US $20.02) digital gift voucher for their time.

### Analysis Approach

Recordings of the interviews were transcribed and combined with the researcher’s notes from each interview. A pseudonym was assigned to each participant to ensure anonymity. We analyzed the data using the 6-phase approach to RTA by Braun and Clarke [40]. The lead researcher (RMK) conducted the analysis as follows.

Phase 1 involved familiarization with the data. RMK read through each transcript twice using Microsoft Word (Microsoft Corp). RMK highlighted passages that were relevant to the research questions and used the comment functionality to capture potential codes and thoughts about the data [43]. In phase 2, RMK imported the data into NVivo (QSR International) and coded each interview using an inductive, data-driven approach. RMK coded the interviews at the sentence level and created codes to capture key ideas. Some codes were semantic (ie, those that captured overt ideas), whereas others were latent codes (ie, those reflecting the researcher’s interpretations) [41]. An example semantic code was *peace of mind*, which was a phrase used by participants, whereas a latent code was *using video calls to monitor health*. Consistent with other examples of RTA [43], we made no attempt to prioritize either form of code. RMK revised and refined the names of the codes as he progressed through the data and used annotations within NVivo to capture additional reflections. Textbox 1 provides an example of a coded data extract [56].

In phase 3, RMK assembled the codes into initial candidate themes by manually grouping codes using NVivo and sketching thematic maps using pencil and paper. RMK discussed the evolving analysis with author YX, who also read through the coded data, sense-checked interpretations, and reviewed the candidate themes.

In phase 4, RMK refined the themes and discussed them with author JW. The authors felt that an initial “benefits and challenges” framing could be helpful to capture the essence of participants’ experiences. This discussion resulted in 6 initial subthemes related to benefits and 5 subthemes related to challenges. In phase 5, RMK defined and named each theme, selecting participant quotes and data extracts to illustrate key ideas. Phase 6 involved writing an initial draft of the report, which involved further theme refinement [41].

After receiving feedback and peer reviewers’ comments on the report, RMK went back through phases 3 to 6 and revised the themes to develop primary interpretations and address the research questions. We also changed our framing to focus on the continuation of care, removing the initial ideas regarding benefits and challenges. Returning to earlier phases in this way is consistent with the tenets of RTA, which champions iteration and argues that analysis “becomes increasingly recursive” [45] as the interpretation progresses. RMK and JW also reviewed the themes to consider whether each one was sufficiently anchored to a central organizing concept [42]. Finally, RMK created a table to describe how the analysis activities align with guidance on establishing trustworthiness in the 6 phases of thematic analysis (Multimedia Appendix 2 [57]). This was done to provide evidence of a trustworthy and credible analysis [58].

From this iterative process, our analysis converged on the central interpretation of video calling as enabling the continuation of care during lockdowns. That is, video calls were not simply a medium for social connection but were being used to continue care practices that would ordinarily take place during face-to-face visits. We created one theme to capture uses that spoke to continuation of care and a second theme to highlight how video calling extended care. We developed 2 further themes: one that interprets organizational issues as impediments to continuing care through video calling and another that highlights the need for 2-way communication for continuation of care. The *Results* section details these themes after presenting demographic data on the participants.

**Example data extract from the interview with participant 7 (Gloria) and codes associated with the extract.**

**Data extract**

- RMK: How do you think going on the video calls helps with that?
- Gloria: I don’t really know that it does help him, but it certainly helps me because I’m reassured seeing that he’s all right or not too bad. And I have noticed particularly, if I don’t see him, if the video call doesn’t come about, then I go in [to see him] and I go, “Oh, he’s gone downhill.” So, it’s more noticeable that way. It’s very, very lonely for him being in there, he’s really not communicating with other residents because of the PSP. So, he’s just either stuck in his room or sitting, looking out of the window in the lounge areas.

**Codes**

- Feeling reassured by video calls
- Importance of visual information and seeing the resident
- Concern about resident decline
- Resident loneliness
- Communication impairment
- Family members are concerned for residents’ emotional well-being

## Results

### Participant Characteristics

We recruited a total of 18 participants (n=15, 83% women and n=3, 17% men) between the ages of 20 and 76 (mean 48, SD 17.3) years. Table 1 lists the participants using pseudonyms along with the relatives they discussed and the technologies they used for the video calls.

Of the 18 participants, 17 (94%) participants lived in Australia, in the state of Victoria, and 1 (6%) participant (Margaret in Table 1) lived in New Zealand but was an expatriate who used video calling to contact her parents in an Australian care home. All had experience living under lockdown conditions in their respective places of residence.

Participants discussed using video calls with a total of 22 relatives, all of whom lived in residential aged care. Of the 22 relatives, 17 (77%) were living in Australia, and 5 (23%) were living in care homes abroad (n=2, 40% in the United States; n=2, 40% in Japan; and n=1, 20% in Italy). The relatives’ ages ranged from 69 to 98 (mean 84, SD 7.3) years. Of the 22 relatives, 18 (82%) were described as having at least one health condition or impairment that affected their use of video calls (Table 1). All the relatives had endured periods of lockdown or isolation at the care homes in which they lived.

All participants had been using video calling with their relatives living in aged care during the pandemic. However, video calling was a new activity for 44% (8/18) of the participants. These individuals had previously visited their relatives in person but turned to video calls during the lockdowns. The remaining 56% (10/18) of the participants had some previous experience using video calling with their relatives in care before the emergence of COVID-19 but became solely reliant on the technology during lockdown periods.

**Table 1:** Participant characteristics and details of the relatives they discussed. All names are pseudonyms.

Pseudonym	Gender	Relative or relatives discussed	Relative’s age (years)	Relative’s impairments affecting use of video calls	Video calling software used	Video calling hardware used
Alison^a^	Woman	Mother	80	Advanced dementia	Zoom	iPad
Ben^b^	Man	Father	83	N/A^c^	Facebook Messenger	Kindle Fire
Charmaine	Woman	Father	75	PSP^d^, nonverbal, and slightly deaf	FaceTime and Zoom	Laptop and smartphone
Deborah^b^	Woman	Mother	86	Advanced dementia	Zoom	iPad
Elaine	Woman	Father	69	Left-side paralysis because of stroke	Facebook Messenger	Laptop
Fiona^b^	Woman	Mother	78	Moderate dementia	Facebook Messenger	Smartphone
Gloria^a^	Woman	Husband	82	PSP	Zoom	iPad
Hannah	Woman	Grandmother and grandfather (married)	75 and 84	Vision impairment and noticeable cognitive decline (grandmother)	WhatsApp	Smartphone
Irene^a^	Woman	Mother	90	Dementia	Zoom	iPad
Jackie^a^	Woman	Mother	88	Advanced dementia, nonverbal, and cannot use her hands	Zoom	iPad
Katherine^a^	Woman	Mother	98	Deafness and vision impairment	Zoom	Laptop, iPad, and smartphone
Luca^a^	Man	Father	90	Moderate dementia and frail with limited mobility	FaceTime	iPad and smartphone
Margaret	Woman	Mother and father	86 and 87	Dementia (mother) and cognitive impairment and memory problems (father)	Zoom	Laptop
Nicole^a^	Woman	Mother	92	Dementia, nonverbal, and limited mobility	Zoom and FaceTime	Laptop and smartphone
Olive	Woman	2 great-aunts	91 and 94	N/A	FaceTime and Facebook Messenger	Smartphone
Paul	Man	Grandmother	84	Deafness and rheumatoid arthritis	Zoom	iPad and smartphone
Quinn^b^	Woman	Grandmother and grandfather (married)	74 and 77	Deafness (grandmother)	Skype	Laptop
Robin^a^	Woman	Grandmother	87	Advanced dementia	FaceTime	iPad and smartphone

^a^Denotes that video calling was a new activity adopted only after the onset of the COVID-19 pandemic.

^b^Denotes that the participant’s relative or relatives lived in an aged care facility outside Australia.

^c^N/A: not applicable.

^d^PSP: progressive supranuclear palsy.

### Theme 1: Video Calls as Enabling the Continuation of Care

#### Summary

This theme responds to our initial question of how video calling was used by family members. The central idea developed through our analysis was that video calling enabled family members to continue participating in the care of their relatives during lockdowns. In other words, video calls provided a way to maintain some of the activities that typically occur during a face-to-face visit. Specifically, participants discussed using video calls for social enrichment and for monitoring residents’ health—both of which would ordinarily take place in person. These uses can be interpreted as relevant to the provision of care—one is about providing social and emotional care, whereas the other is about monitoring physical and mental well-being. Both are about ensuring that the resident is being “cared for” in an appropriate way.

#### “It’s So Good to See Them”: Using Video Calls to Continue Social Enrichment

The first aspect of continuing care was demonstrated through participants’ descriptions of providing social enrichment when physical visits were not possible. These accounts were often couched in the need to prevent “decline.” Gloria, for example, discussed the welfare of her husband, who had moved into care because of progressive supranuclear palsy. She said that her husband had become “very lonely” during the lockdowns. Therefore, video calling was her husband’s primary source of social enrichment, and Gloria felt that there was a clear and noticeable impact on his well-being if video calls did not occur. Upon visiting him in person between lockdowns, she observed the following:

I have noticed particularly, if I don’t see him, if the video call doesn’t come about, then I go in [to see him] and I go, “Oh, he’s gone downhill.” So, it’s more noticeable that way. It’s very, very lonely for him being in there, he’s really not communicating with other residents because of the PSP. So, he’s just either stuck in his room or sitting, looking out of the window in the lounge areas.

Participants described diverse uses of video calls for the continuation of social enrichment. In some cases, video calls were used for short “catch ups” and “chit-chat,” which provided opportunities to share updates about happenings outside the care home, keeping the resident involved in family life. Other cases involved the use of video calls for in-depth relationship maintenance. Quinn described how Skype calls with her grandparents would typically last over an hour and would involve different activities:

I always use my laptop when we Skype and I share my screen when I want to show them something. It’s always good to show them photos of everything while we are Skyping so that we can talk about them together. And we also watch videos together sometimes.

Quinn’s quote shows how video calls were used to mediate intergenerational activities that constitute an enriching social life and that would otherwise be impossible during lockdowns without using technology. Video calls were also used to continue with other events and social situations. Katherine described how, when it was her mother’s birthday, she was able to visit the facility between lockdowns and use a video call to host a celebration with extended family members:

When it was her 98th birthday in June (2021), I took my laptop in...Then my brother and I had the laptop and we Zoomed my other brother. We had him sort of in the background, chatting away while we had a birthday cake and some of the staff came in with more cake.

Although these findings reinforce the notion that video calls can support social connections between family members and care home residents [28,31,59-61], we interpret them as the continuation of care. That is, family members wanted residents to feel cared for and know that someone cares about them. This was reinforced by descriptions of the positive emotional outcomes that arose from video calls and how they benefited residents:

I have a four-year-old son, so I try to get him to say hello too, which always puts a smile on dad’s face...then if I’m at home with my son, I can put him on and my husband and we can all say hello.

Charmaine

[Dad] would be happy and smile and be positive about having received our calls. It’s good for him to see us and remember us, and to see my mother and talk to my mother and me and my brother.

Luca

#### “It’s Important to Have My Own Sense of His Health”: Using Video Calls to Continue Health Monitoring

The second aspect of continuing care involved family members using video to monitor the health of their relatives. Outside of a lockdown, such monitoring might naturally occur as part of a physical visit. The fact that this took place through video calls further evidences their use for continuation of care.

For participants such as Jackie, monitoring her mother’s health was a key motivator for adopting video calls during lockdown. She felt that information from care staff was useful but not entirely sufficient, and she expressed a need to do “checking up” on her mother:

When we went into lockdown, we couldn’t see how she was. She can’t talk to us. Even though the nursing staff will give us some information, you still want a little bit of checking up, if you know what I mean? So we started to have Zoom meetings with her.

Participants described making inferences about their relatives’ health during video calls based on visual appearance and the tidiness of their living environment. These actions can be understood as constitutive of care given that they represent attempts to determine whether something is wrong and, hence, whether intervention is required. Olive described how she used video calls to check for what she called “signs of deterioration”:

I’m attuned to looking for particular things. Have they done their hair? Have they done their nails? What’s their environment like? Are they taking care of themselves? Are their clothes clean? Those kinds of things are very important to me. That’s part of me knowing that they’re in good health, because that’s how they normally present themselves.

A participant, Ben, provided a compelling example in which he used a video call to infer that his father had contracted COVID-19. His father had previously tested negative for the disease following an outbreak within his facility, but interactions during a video call convinced Ben that further action was required. The following extract reveals how Ben’s intervention led to his father receiving treatment and emphasizes the role of the video call in making his assessment:

I noticed things were off in his voice. And I just kept asking him, “Are you okay?” And it finally led to me saying [to the staff], “I think you need to test him again.” I just got the sense that he wasn’t well. Based on how he looked, how he was physically presenting on the video, as well as how he sounded. And don’t take the audio too far on that one, because I think a lot of it came from his visual appearance. He just doesn’t look well. They wound up finally having a closer look and he was eventually taken to the hospital and treated

for COVID-19

In addition to monitoring health and well-being, Ben’s quote alludes to the role of video in enabling participants to advocate for care to be provided. In this case, Ben contacted the staff via telephone and requested additional care. Another participant, Fiona, described how video calls enabled discussions with other family members when monitoring the health of her mother, who was living with dementia:

Having the video is good, especially for her sake. I can see her health and how she’s behaving. Because my brother will say, “Her memory’s not good. She’s kind of spacey,” and it’s one thing to say it, but then for me to actually see her and how she behaves, it’s a lot easier.

Here, Fiona used video to verify information obtained from other people—in this case, her brother—when maintaining an understanding of her mother’s health. Video calls also provided the opportunity to speak with nursing staff at the care homes. Gloria described how she would often be able to speak to staff during the video calls. This helped relieve anxiety about her husband’s well-being:

I can ask them questions about how he’s been, “Is he depressed today? Because he has been quite depressed.” And they can reassure me or say, “Look, talk to him for a few minutes.”

Using video calls in this manner helped participants alleviate feelings of anxiety and provided “peace of mind.” For example, Olive told us that using video enabled her to feel reassured about her aunt’s welfare when she could not visit:

I was able to physically see in the room, and I was able to make sure that she had the things that she needed. Now, she was fine, but the peace of mind it gave me that the room was clean, that her personal effects weren’t being interfered with, that she had the basics...Those sorts of things are really important to me.

These examples illustrate how video calls provided opportunities to continue care practices that would ordinarily be conducted face to face and that might have been difficult or impossible when in-person visits were restricted.

### Theme 2: The Role of Video in Extending Care

This theme responds to our initial question of how video calling was beneficial to family members, that is, how specific qualities associated with using video calls contributed to the continuation of care. This means emphasizing the value that video calls add to care—not just how video calls contributed to the continuation of care but how they enhanced care as well.

#### “I Get to Speak to Her More Than Before”: Video Calls Enabled Increased Frequency of Contact

The availability of video calling enabled some participants to contact their relatives more frequently after the pandemic began. Several participants (8/18, 44%) lived far from their relatives’ care homes and, hence, incurred substantial costs when visiting. These participants described visiting once per week or several times per month before the lockdowns. The transition to video calling allowed these participants to “visit” more frequently than before. Olive, for example, commented on how the frequency of contact with her great-aunts had increased after the lockdowns and how it involved extended family members:

In terms of frequency, I would visit once a month on a Sunday [before the pandemic]. During COVID we would FaceTime once a week...That was done as a family, as well. We would have two- or three-way conference calls.

Although face-to-face visits were still preferred, the ability to be in more frequent contact was important to those who were concerned about their relative’s health. Ben, for example, valued the ability to pay more attention to his father when there was an outbreak of COVID-19 at his facility. However, some participants (2/18, 11%) mentioned that facilities had discontinued support for video calling when the lockdowns were lifted as they viewed it as nonessential. These individuals wished that video calling had continued so that they could maintain the increased frequency of contact. Gloria, for example, commented on the inconsistent availability of video calling at her husband’s facility:

Once lockdown is over, that’s the end of the video calls and it would be so much better if they could keep it going. And then, I could just touch base with him every day to see how he’s going and he knows that I’m there and caring for him.

This extract speaks to frequent contact as a means for families to convey the sense that the resident is being cared for. Although increasing the frequency of contact does not guarantee higher-quality interactions [62], our participants’ experiences speak to the value of having a communication channel available and highlight the importance of being able to contact a family member during times of distress.

#### “It’s Crucial for Us to See Him”: Video Provided Access to Nonverbal Cues

Many participants’ experiences stressed the importance of the visual layer associated with a video call. Being able to see the resident is a key differentiator between video and other communication technologies such as the telephone.

We interpreted participants’ experiences as signaling the importance of nonverbal cues for the continuation of care. Such cues include gestures and body movements, facial cues such as lip and eye movements, and the tone of voice. Previous work has argued that nonverbal cues contribute to the “richness” of video calls and enhance feelings of social presence [30,57,63], which refers to the salience of the other person in the interaction [64]. Higher social presence is thought to promote a sense of togetherness when using video calls [18,59].

Our analysis of participants’ experiences suggested that transmitting nonverbal cues played other roles in providing care. First, nonverbal cues were crucial for participants whose relatives had severe communication impairments. These people emphasized that it was essential to *see* their relatives when communicating with them, which made video calling more helpful than modalities such as the telephone. As an example, Charmaine discussed how nonverbal cues were crucial for interacting with her father, who had become largely mute because of progressive supranuclear palsy. She said the following:

...he will mouth words, but he doesn’t generally speak out loud and he has trouble finding his words. So we’ve really found that video conferencing works best so you can get the non-verbal cues.

When asked to elaborate on what kinds of cues are important and how they help, Charmaine said that nonverbal cues were useful for the following:

...being able to see that he’s thinking, because you can see where he’s looking often...And I guess we’re getting better at reading his body language too. He tends to nod, or shake his head, or mouth the words “no” or “yes,” or he’ll just shrug. We also prompt him to speak out loud which used to work more effectively than it does now. But sometimes he can come up with a sentence, and I’ll put my hand up. So I’ll use nonverbal cues as well, like putting my hand up [to show] that I can’t hear.

Charmaine’s quote points to the role of eye gaze direction, speed of response, and body language when conversing with her father. It also reveals that the utility of nonverbal cues is bidirectional, with Charmaine using her own gestures to convey information back to her father. These interactions illustrate the complexities of communicating with people living in aged care and highlight the additional value that video-mediated exchanges brought during the lockdowns.

A second role of nonverbal cues was to support the conveyance of warmth and affection during emotive communication. Deborah recalled the importance of gestures when interacting with her mother, who has dementia:

Gesture communication is very important, especially being Italian. When on the video call, we send kisses to each other, not only at the end, but during the communication itself. I always try to smile to her, she recognizes it. Sometimes I sing songs to her. So it’s not only verbal communication but many other things. I showed her a soft toy and she started laughing. It was funny, she reminded me of a little girl smiling to a doll. It works, somehow, I feel that she is there. She is in a connection with me, a member of the family again.

The importance of nonverbal cues also came to the fore when discussing the widespread use of face masks during the pandemic. Several participants (6/18, 33%) were able to visit their relatives in between periods of lockdown, when bans on visitors were lifted temporarily. These visits required the use of face masks to comply with government regulations. Participants reported that masks made conversations difficult as they occlude facial cues that can underpin nonverbal communication. Masks also made it difficult for residents to understand what was being said, especially among those who were hard of hearing. The fact that video calls did not require masks was said to make conversations “closer to normal.” For example, Elaine told us the following:

One difference between face to face and video is that we are not wearing our masks on video calls. When we go there we have to wear a mask in person. So we have these face shields, and that means it is more natural on video calls. It doesn’t seem like there is so much of a barrier, even though we’re not physically there, it just feels like a barrier, a perceived barrier, when we are wearing masks.

Taken together, the availability of nonverbal cues can be seen as making a video call feel more like a physical visit compared with wearing a mask or using a modality such as the telephone.

### Theme 3: Organizational Constraints Threatened the Continuation of Care Through Video Calling

This theme brings together organizational issues that negatively affected participants’ use of video calling. Our interpretation is that these issues were problematic as they hampered participants’ ability to enact continuation of care.

#### “There’s Problems With the Connection There”: Video Calling Was Hampered by Limited Technology Infrastructure

Consistent with prior work [33,34], the lack of high-quality technology infrastructure in care homes was a problem with video calls. Participants reported that their relative’s aged care facility did not have reliable internet. This led to mixed experiences and unpredictable call quality. Margaret shared the following:

Some weeks, we’ll have a beautiful conversation and it doesn’t drop out at all. Then, more often, we’ll have parts of the conversation where we freeze or they freeze. Sometimes they drop-off completely.

These connectivity problems led to anxiety and stress for residents and sometimes caused them to think that they had “broken” the technology. Participants described having to spend time reassuring relatives in this situation and had to fix technical problems over the telephone. This was not always easy without the ability to visit in person.

In addition to the lack of stable connections, it became apparent that many homes had a limited pool of technologies available to support video calls. Katherine, for example, said that her mother’s aged care home had “only one iPad for 63 families.” This meant that video calls were sometimes cut short as the device was needed for other residents. In some cases, staff members tried to overcome this problem by loaning their personal devices to residents. However, it seemed that the loaning of devices further increased the likelihood of calls being cut short. Robin, who had been using video to see her grandmother, felt that this made some video calls “rushed and inauthentic.” She said the following:

...the worst part is when they cut you off...if they use their personal phones, it’s like, “Oh, sorry, I have to take this, there’s something urgent I need the phone for.”

We interpret these experiences as limiting participants’ ability to carry out actions relevant to continuing care. That is, poor connections and rushed calls impeded the ability to continue care by providing insufficient information about the residents’ well-being.

#### “It Was Like Calling a Prison”: Video Calls Had to Be Booked in Advance

A putative benefit of video calling is that family members can potentially make and receive calls at any time and at their own convenience. However, the interviews revealed that this benefit was not realized for all participants. Instead, the lack of technology in some homes meant that video calls had to be scheduled in advance. This was done using booking systems created from an assemblage of web-based calendars, email, and telephone. There were also cases where video calls were available only during restricted “visitation hours”—such as at fixed times in the morning, afternoon, and evening. This meant that participants had to schedule their days around these windows, and if they could not be available at the specific time, they were not able to see their relatives.

The need to schedule calls was a repeated source of frustration and became a barrier to continuing care. Nicole, for example, described it as “a pain” and wished video calls could be used similarly to telephone calls as “they [the home] allow you to ring anytime.” The restrictions around call times, together with the lockdown conditions in care homes, were described as creating a “prison-like setup.” Irene said that the following occurred during one of the lockdowns:

You could not ring anybody in the facility, nor the front desk. It was like a prison, they had one number you could ring and then you could organize something and so you would send an email and say, “Can I have the appointment to Zoom at this time?” And if that one worked, then we could say, “Same time tomorrow, please.” And they’d pencil it in.

Despite participants’ attempts to work around the schedules imposed by the care homes, there were cases in which prearranged calls were late or failed to materialize, causing frustration. Gloria told us the following:

[the home] might set up a time, but they’re never on time. So, you have to be available for, let’s say, a frame of two hours. I keep telling myself, just be patient.

Finally, there was considerable variation in the duration and frequency of calls. Gloria stated that one lockdown involved “two calls a week set up by the staff, but five-minute duration,” whereas Alison said that calls lasted “half hour or a 15-minute session.” These calls were the only source of outside social contact for the residents. Although these short calls may be better than no contact at all, they should raise questions about the adequacy of solutions provided during the pandemic and how these might be improved in the future.

#### “The Staff Are Flat Out Now”: Staff Involvement Was Needed but Was Often Scarce

Consistent with prior work [36,37], participants raised the need for staff to sometimes assist residents with video calling. Staff were involved in making calls, manipulating the hardware and software, and fixing technical issues. Staff were seen as essential for supporting video calls with residents who had complex care needs, especially those living with dementia. In this case, staff were needed for actions such as directing the resident’s attention to the call, explaining who the resident was talking to, and repeating phrases and words if residents could not hear the video call properly. However, staff were reported to be time-pressured and not always available to assist with these activities.

The need for staff involvement was especially challenging during the COVID-19 lockdowns because of staff shortages and outbreaks within the aged care sector [65]. Some participants (5/18, 28%) felt that staff turnover was a problem that affected video calling. These individuals had developed co-operative relationships with specific staff members. Video calls were reported to be difficult or impossible when these staff members were absent. For example, Ben said the following:

Like most aged care facilities, they’re at their capacity to provide health assistance. And so it took a while to identify a couple of the nursing staff who both are willing and can find the time to actually help him do calls. And so far, the only ones we’ve been able to do is when I’m calling him and they answer for him.

### Theme 4: Successful Continuation of Care Required 2-Way Communication

This final theme highlights 2 issues that further impeded continuation of care but that focus on the need for the people in aged care to be able to use and respond to video calls.

#### “She Was Never What You Would Call Tech-Savvy”: Some Residents Were Unfamiliar With Video Calls

Some participants (6/18, 33%) stated that their relatives in care were unfamiliar with video calls and had received no training on how to use them. This was a challenge as their relatives did not know how to operate the software and hardware independently, meaning that calls failed to happen or went unanswered.

Participants also mentioned that their relatives did not always understand the concept of positioning oneself in the camera feed. This led to situations in which the participants saw their relatives from awkward angles or without their faces in the frame. Gloria said that her husband did not understand how to manipulate the iPad provided to him at the care home. She recounted the following:

[He] just never had anything to do with computers or really technology at all. He didn’t get the concept that you can see yourself on the screen if you’ve got it aimed correctly. That made it rather difficult.

To overcome this problem, Gloria and other participants noted that staff involvement was needed. However, as noted previously, this was not always possible. She said the following:

[The staff often] go away and leave him, and I would end up looking at the ceiling because the iPad would fall over or something like that, and all I could see was the ceiling.

This challenge can be interpreted as further affecting participants’ ability to continue care, especially in monitoring health. This is because the underlying concern relates to acquiring visual cues; when the camera is not angled correctly, it becomes impossible to make inferences about the residents’ welfare.

#### “For Her, This Could Be a Broken Mirror”: Residents’ Health Conditions Make Video Calls Challenging

Many people in aged care are living with profound health challenges, some of which precipitate their move into care [66,67]. Participants reported that residents’ health problems affected their ability to participate in video calls, often because commercial systems are ill-suited to the aged care setting [35] and require adaptation to be successful [61].

For example, Paul discussed how his grandmother had arthritis in her hands, making it difficult for her to use computers for video calls*.* To overcome this issue, Paul and his family purchased a large-screen smartphone and “jacked up the size of the keyboard” to make the device more usable for her.

More challenging cases were reported by participants whose relatives had moderate or advanced dementia. Some (4/18, 22%) stated that, although video calls were valuable for checking on their relative’s well-being, the conversation was limited as the relative did not fully understand the nature of video calling technology. Our first participant, Alison, had been using video calls to see her mother but decided to abandon them as her mother could no longer comprehend what was happening. She said the following:

Sadly, because my mum had dementia, she couldn’t actually use the technology. It didn’t work for her, so we essentially got cut off from her for 18 months. In that way, they made it an option, but it wasn’t an option for someone with [advanced] dementia.

When asked to elaborate on why the technology “didn’t work,” Alison discussed how her mother became confused when seeing an image of herself on screen, which is a common feature of video calling applications such as Skype and Zoom. Alison said the following:

My mother couldn’t understand what was going on. She wouldn’t interact with it. She wouldn’t look at the screen. If she saw herself on it, she’d just be staring and trying to figure out who that person was. She was better at engaging in person.

This example highlights that, although family members may still obtain some benefits from seeing relatives through a video feed, different approaches may be required to enable families to continue seeing residents who have advanced care needs during times of social distancing.

## Discussion

### Principal Findings

This study aimed to understand family members’ uses of video calling during lockdown restrictions in aged care. The main finding of our analysis was that video calling became a medium for family members to enact the continuation of care during lockdown restrictions. Although participants’ experiences of video calling were complex and multifaceted, there was an underlying goal of using the technology to continue care and ensure that care was provided. However, the use of video calls was affected by organizational constraints and the need for 2-way communication with relatives, which was often difficult. These findings contribute to an improved understanding of why video calls were valued during the pandemic lockdowns and extend the knowledge of the challenges that families encountered [33].

Previous research on the use of video calls in aged care has focused primarily on the potential to support social interaction and the technological barriers that can affect their use. Our study is the first to suggest that video calling systems are used for the continuation of care when families cannot visit the facility. This interpretation moves beyond existing characterizations of video as a tool for social connectedness [27] to one in which video calls are used to continue care practices when physical visits are restricted. Many of the activities conducted by participants in this study, including relational maintenance and health monitoring, align with the findings of ethnographic studies on the coordinated work of families in care homes [67]. Our analysis suggests that, when video calls were used to replace in-person visits, they became a modality for families to continue these contributions to care. However, they can also enable new kinds of interactions, such as sharing digital content over distance and supporting meetings with extended family. This speaks to the potential for video to enrich caregiving in ways that go beyond what is possible in a face-to-face visit.

This study also suggests that video calling supports family members’ own peace of mind. The COVID-19 pandemic was an extremely stressful time for families, and it has been suggested that the inability to visit during lockdowns produced feelings of helplessness and anxiety [9]. Concerns about residents’ health may have been especially salient given the high rates of mortality in aged care [65], leading family members to request more frequent updates about residents’ welfare [11,68]. However, video calling can convey information that provides peace of mind without placing additional burden on staff members to provide such information. This raises the importance of video calling from a platform of conversation to one that can enhance care in multiple ways, emphasizing the need to improve provision of video calls and develop solutions that are better suited to the aged care context. Our findings highlight the urgent need for video calls to be seen within the aged care sector not just as a secondary means of providing social support but also as a vital tool that allows families to contribute to the ongoing care of their loved ones.

In addition to continuing care, our analysis indicates that video calling can enhance family members’ caring practices when forced to see their relatives from a distance. When video calling worked well, participants were able to have more frequent contact than before and were able to access nonverbal cues that were essential for communicating with residents who had impairments. This finding is important as, in some countries, care homes are mandated to provide telephone communication with relatives [13], but video calling is an optional extra. Providing better support for video calling can help families whose relatives have hearing and speech impairments and who depend on gestures and lip movements to understand what is being said. This also speaks to the value of supporting video calling outside of lockdown periods for family members who cannot easily visit the care home [28,58].

Finally, our analysis identified that the continuation of care through video calling was impeded by multiple challenges. One problem relates to the constraints of the aged care context, where homes are often underresourced and understaffed [67]. This was especially challenging during the pandemic [65] and influenced the quality, reliability, and length of video calls. Residents’ unfamiliarity with video calling and their health problems were additional barriers that affected the success of video calls [35,37]. Given the important role that video calls can play in the continuation of care, addressing these challenges can no longer be seen as a secondary concern that is relegated to the periphery of lifestyle programs but must be seen as central to supporting the ongoing welfare of aged care residents.

### Comparison With Prior Work

The notion of video calling as a medium for the continuation of care aligns with ideas from early studies that used videophones, which were a primitive form of video calling technology. Demiris et al [60] suggested that videophones could support “distant caregiving.” Our work substantiates and adds weight to this claim by showing how the high-fidelity feeds of modern systems can be used in the practice of continuing care. We also emphasize the ability of video to support caregiving during periods of lockdown, which may become more common in the future if COVID-19 becomes endemic [22] and because of seasonal variation in other viral diseases [69].

The challenges identified in this study align with findings of previous trials of video calling in aged care. Examples include limited technology literacy among some residents, low device accessibility, poor Wi-Fi, the lack of devices in care homes, and the need for staff to help with video calls [30,34,35]. In addition, our findings resonate with a study conducted in Canada during the COVID-19 pandemic [33]. Similar to our work, the Canadian study found that care homes had very few devices to facilitate video calls, that video calls were often missed and late, and that staff lacked time to help with facilitation [33]. Taken together, these findings suggest that there are systemic issues in aged care that need to be addressed to better support video calls and the crucial role they play when in-person visits are not possible.

A novel finding of this study was that resource constraints required care homes and families to coordinate call times using booking systems, which may have also helped ensure fair and equitable access to limited devices. However, this coordination was effortful for family members and led to unsatisfying interactions when calls were late or missed. It is likely that this issue was not documented in previous studies as they did not examine the use of video calling during periods of lockdown. This addition to the literature is important as it reveals a negative experience for families and residents that should be avoided during future lockdowns in care homes.

### Implications for Video Calling in Aged Care

This study highlights that ongoing support for video calling in aged care is essential. We argue that video calling should, therefore, be elevated to the status of critical infrastructure given its potential to enable the continuation of care and mitigate the negative consequences of enforced isolation.

However, video calling needs more infrastructural support in care homes. It must be recognized that social connection is an essential human right and must be supported as a basic activity. Policy makers should recognize that staff may need additional time to assist with video calling. Better resourcing is needed for technologies that can be shared among residents at times when face-to-face visits are not possible, and aged care workers should not have to use their own devices to support the social well-being of residents.

The problem of coordinating video calls between families and residents should also receive attention in terms of improving residents’ freedom to make and receive calls during lockdown. If video calls are used at times when in-person visits are prohibited, there is a need for care providers to consider ways of ensuring that calls can happen on a regular basis and to support interactions that go beyond mere glimpses of the resident. Although short video calls may be better than no contact at all, they are unlikely to constitute sufficient social contact and can create anxiety among families. This can also be seen as an equity issue in that being able to see a loved one should be a right, not a privilege.

Improving the design of video calling systems for aged care can also help lower the burden placed on staff when it comes to administering and running video calls. Our findings reiterate that commercial video calling systems can be hard to use for care home residents [35,59]. To provide a suitable user experience, future designs should aim for the ease of use, learnability, and accessibility of software, especially for those with dexterity impairments [70,71]. Video calling hardware could also be made more suitable for aged care, such as by using physical stands or supports that enable residents to maneuver the video calling device into a comfortable position without needing substantial involvement from caregivers [61]. An ideal video calling system for aged care would be accessible and support independent use, incorporating software that is easy to understand and hardware that can be positioned according to the resident’s needs. This, in turn, would reduce the need for staff involvement.

### Strengths and Limitations of This Study

This study has 2 main strengths. First, our analysis is grounded in real-world experiences of video calling deployment during lockdowns. This is different from most previous studies, which typically involved participants who were given a technology to trial by a research team [28,31,32,66]. Our findings reflect the experiences of families and care homes who had to manage the technology by themselves during unexpected and indeterminate periods of lockdown, evidencing the issues that arise in this situation.

The second strength of this study lies in our focus on close family members, many of whom played an important role in caring for their relatives. Previous studies have often been designed to test the feasibility of using video calls to initiate connections with family members who do not typically visit the home (eg, distant grandchildren who play no substantial caring role [28]). Our work expands the scope of the literature to provide an understanding of why video calls are valuable to family members who play considerable caring roles.

The main limitations of this study are 2-fold. First, the focus on family members does mean that we excluded the views of aged care residents and staff on video calling during periods of lockdown. We were not able to interview these groups as care facilities were inaccessible during data collection. Future research should address this gap. Second, this study focused solely on the experiences of people living in Australia and New Zealand. Aged care systems in other countries may have procedures in place to better support video calling during lockdowns, although as we have noted, a Canadian study highlighted similar concerns [33]. Future studies that compare video call experiences in diverse aged care contexts and in other countries are warranted.

Finally, trustworthiness is an important consideration when evaluating the credibility of qualitative research [58], but there is an open question as to what makes RTA trustworthy. We consider our analysis trustworthy as it matches well with guidance for producing trustworthy thematic analysis [57] and for high-quality reflexive analysis [45]. Examples of practices that evidence trustworthiness in our approach include a clear rationale for using RTA [45], prolonged engagement with the data [72], and full disclosure of our analytic process [57] (Multimedia Appendix 2). To improve our practice in the future, we recommend using a reflexive journal to document the analytic process [73] and create an audit trail [57].

### Conclusions

This study investigated how family members used video calls with older adults in residential aged care during lockdowns arising from the COVID-19 pandemic. Overall, our findings suggest that video calls were used by family members to continue care practices at times when face-to-face visits were not possible. Families were able to provide social enrichment and monitor the residents’ health and well-being, which they could then use to ensure continuity of care. Video calls extended care by enabling frequent contact and were crucial for those who were reliant on nonverbal cues for communication, sometimes making them preferable to physical visits in which masks were required.

However, this study shows that family members’ ability to engage in the continuation of care was hampered by organizational issues, particularly the lack of digital technology in aged care. Although this can be partly attributed to the speed at which care homes needed to adapt and to the loss of staff during the pandemic, the situation revealed the paucity of support for video calling in many care homes. If video calls are to be used to mitigate social isolation arising from infection control measures, there is an urgent need for better provision of technology and improvement of infrastructure to enable calling. There may also be a need to allow for staff to support video calling, especially for residents who cannot operate the technology independently.

To conclude, our findings emphasize that governments and aged care providers should fully support the implementation of video calls between residents and their family members during times when social distancing is required. This study also reiterates the need for video calling solutions that are better suited to aged care such that families can remain connected with residents even when they are forced to be physically apart.

## Appendix

Multimedia Appendix 1 Interview questions used in the study.

Multimedia Appendix 2 List of techniques used to establish trustworthiness in our analysis based on the Means of Establishing Trustworthiness by Nowell et al [57].

## Abbreviations

RTA: reflexive thematic analysis

## Data Availability

The data sets generated and analyzed during this study are not publicly available as ethics approval was not given for the data to be made public. Requests to access the data sets should be directed to RMK. The data may be made available upon reasonable request.
