# Supplementary material for: Video Calls as a Replacement for Family Visits During Lockdowns in Aged Care: Interview Study With Family Members
Source: JMIR Aging. 2023 Jun 12;6:e40953. doi: 10.2196/40953 (PMC10263180; doi:10.2196/40953)
Supplement: Multimedia Appendix 1 [file aging_v6i1e40953_app1.doc]

Appendix I

Demographic Questions

A few questions about you:

- Which city and state is your main place of residence?
- What is your employment status? Full time, part time, student, unemployed?
- May I ask your age and gender?

Now a few questions about your relative:

- Who is the relative that were you planning to talk about today?
  - Which country are they living in?
- What kind of care facility do they live in?
- May I ask their age?
- Are they living with any health conditions that might be relevant to the conversation today?
- Is it okay if I ask questions about that health problem during the interview?

Semi-Structured Interview Questions

Introductory Questions

1. Can you start by telling me a bit more about how you've been using video calls to stay connected with your relative?
2. What's the reason you chose that technology to keep in contact with them?
3. What have the main benefits been for you in terms of using that technology?
4. What about your relative, how does it benefit them?
5. Have you faced any difficulties with that technology?
6. You mentioned that your relative has [health problem]. How does that affect their use of the technology we’ve been talking about?

Pandemic-specific Questions

1. What were things like before the pandemic in terms of visiting your relative?
2. What changed once the lockdowns started?
3. How was your relative affected by the situation?
4. How was the technology helpful in keeping connected?
5. What was the reason you used that technology?
6. Were there any challenges in using that technology during the pandemic?

1. Did you receive any help from the aged care provider in terms of using the technology?
2. What has been happening since the lockdowns ended?
3. Is there anything else that we didn’t cover or which you would like to add?
