# Supplementary material for: Video Calls as a Replacement for Family Visits During Lockdowns in Aged Care: Interview Study With Family Members
Source: JMIR Aging. 2023 Jun 12;6:e40953. doi: 10.2196/40953 (PMC10263180; doi:10.2196/40953)
Supplement: Multimedia Appendix 2 [file aging_v6i1e40953_app2.docx]

Table 3. List of techniques used to establish trustworthiness in our analysis, based on Means of Establishing Trustworthiness by Nowell et al. [57]

| **Phases of Thematic Analysis** | **Means of Establishing Trustworthiness (from [57])** | **Relevant Techniques Used in This Study** |
| --- | --- | --- |
| Phase 1: Familiarization | Prolonged engagement with data | RK engaged in multiple rounds of reading and reflection about the data over almost one year, from initial collection through to writing the report (Phase 6). |
|  | Documentation of theoretical and reflective thoughts | RK recorded thoughts using comments in Microsoft Word during initial read-throughs. |
|  | Documentation of thoughts about potential codes and themes | RK recorded potential codes using Microsoft comments, and later using NVivo during Phase 2. |
|  | Raw data stored in well-organized archives | All transcripts named with unique ID number. Text equally spaced with page and line numbers for reference of specific passages. Data stored in an organised file system on a password-protected server. Individual folders for each participant. |
| Phase 2: Generating initial codes | Researcher triangulation | Two authors read the data independently. RK coded the data and YX read through the coded data. |
|  | Audit trail of code generation | All coding conducted using NVivo.  New versions of the project file saved after each round of coding. |
| Phase 3: Searching for themes | Researcher triangulation | Authors RK and YX discussed initial interpretations and potential themes. All authors were involved in discussions about subsequent interpretations and potential framing of the research report. |
|  | Diagramming to make sense of theme connections | RK used pencil and paper sketches to sketch initial thematic maps. |
| Phase 4: Reviewing themes | Researcher triangulation | All authors were involved in discussion of themes. |
|  | Themes and subthemes vetted by team members | Themes discussed in regular research meetings between RK and JW.  Initial framing around “benefits and challenges of video calling” tested and later removed based on feedback. |
|  | Test for referential adequacy by returning to raw data | RK returned to raw data several times to ensure iterated themes were supported by data extracts.  Inclusion of participant quotes in subtheme titles as evidence of fit. |
| Phase 5: Defining and naming themes | Researcher triangulation | RK renamed several times in line with the ongoing analysis.  Co-authors provided suggestions of literature to inform interpretations. |
|  | Peer debriefing | Peer reviewers gave feedback on themes and suggestions for revising the research report. |
|  | Team consensus on themes | All authors provided feedback on themes and reorganisation. Initial “benefits and challenges” framing revised based on reviewer and co-author feedback. JW revised theme names in final version of the report. |
|  | Documentation of theme naming | Themes given names in NVivo and in drafts of the research report. |
| Phase 6: Producing the report | Peer debriefing | The report was revised several times in response to peer reviewers’ feedback, and in collaboration with co-authors.  The final version of the report looks very different to earlier versions. |
|  | Describing process of coding and analysis in sufficient details | The report describes the coding process in detail, following guidance from Braun & Clarke and best practice from previous examples. |
|  | Report on reasons for theoretical, methodological, and analytical choices throughout the entire study | The report includes a Study Design section in the Methods to outline key choices. |
